# Patient throughput times and inflow patterns in Swedish emergency departments. A basis for ANSWER, A National SWedish Emergency Registry

**DOI:** 10.1186/1757-7241-19-37

**Published:** 2011-06-13

**Authors:** Ulf Ekelund, Lisa Kurland, Fredrik Eklund, Paulus Torkki, Anna Letterstål, Per Lindmarker, Maaret Castrén

**Affiliations:** 1 Emergency Medicine, Department of Clinical Sciences at Lund, Lund University, Sweden; 2 Karolinska Institutet, Department of Clinical Sciences and Education and Section of Emergency Medicine, Södersjukhuset, Stockholm, Sweden; 3 Karolinska Institutet, Medical Management Centre, Stockholm, Sweden; 4 HEMA-Institute, BIT Research Centre, Aalto University, Finland; 5 Emergency Medicine, Karolinska University Hospital, Stockholm, Sweden

**Keywords:** Emergency department, Quality measures, Quality of care, Throughput times, Registry

## Abstract

**Objective:**

Quality improvement initiatives in emergency medicine (EM) often suffer from a lack of benchmarking data on the quality of care. The objectives of this study were twofold: 1. To assess the feasibility of collecting benchmarking data from different Swedish emergency departments (EDs) and 2. To evaluate patient throughput times and inflow patterns.

**Method:**

We compared patient inflow patterns, total lengths of patient stay (LOS) and times to first physician at six Swedish university hospital EDs in 2009. Study data were retrieved from the hospitals' computerized information systems during single on-site visits to each participating hospital.

**Results:**

All EDs provided throughput times and patient presentation data without significant problems. In all EDs, Monday was the busiest day and the fewest patients presented on Saturday. All EDs had a large increase in patient inflow before noon with a slow decline over the rest of the 24 h, and this peak and decline was especially pronounced in elderly patients. The average LOS was 4 h of which 2 h was spent waiting for the first physician. These throughput times showed a considerable diurnal variation in all EDs, with the longest times occurring 6-7 am and in the late afternoon.

**Conclusion:**

These results demonstrate the feasibility of collecting benchmarking data on quality of care targets within Swedish EM, and form the basis for ANSWER, A National SWedish Emergency Registry.

## Background

Large resources are used in local and regional initiatives to improve the quality of emergency care. If such initiatives are to be successful, they need to be based on reliable data on the quality of care at the single emergency care center and, for benchmarking, at similar other centers. However, since benchmarking data are often lacking [1], quality improvements are commonly suboptimal and may not represent the best use of the available resources.

Limited benchmarking data relating to emergency care may be obtained from existing multicenter patient databases or registries. However, almost all such registries focus on single disease groups [2-6] or specific medical interventions [3,7,8]. Very few registries focus on the emergency care process and none were primarily formed to reflect the quality of care. For instance, the North Carolina Disease Event Tracking and Epidemiologic Collection Tool (NC DETECT [9-11]) is an emergency patient database with the main purpose of public health surveillance and early detection of large medical events. Another database in the United States (US), the National Hospital Ambulatory Medical Care Survey (NHAMCS [12]), uses a national probability sample of visits to U.S. hospital EDs to produce annual national estimates of ED visits. Results from this database do not apply to individual EDs, and are delayed more than one year which precludes their use for optimal benchmarking. The Quarterly Monitoring of Accident and Emergency (QMAE) [13] in the United Kingdom (UK) receives and publishes aggregated operational data submitted by EDs. The UK Hospital Episode Statistics (HES) [14,15] includes individual patient data but do not include all EDs and are only published every second year. None of the mentioned databases include information regarding mortality and morbidity during or after the ED visit.

The objectives of the present study were twofold. One was to assess the feasibility of collecting selected quality of care data from six different Swedish EDs using automated data capture as a basis for a national quality of care registry, and the other was to present some first results regarding throughput times and patient presentation times. In this paper we present the basis for ANSWER, A National SWedish Emergency Registry.

## Methods

### Study design and setting

This study compared variables reflecting quality targets in the emergency care at six adult EDs in Sweden in 2009; Uppsala University Hospital, Karolinska University Hospital in Solna and Huddinge, Södersjukhuset in Stockholm, Sahlgrenska University Hospital in Göteborg and Skåne University Hospital in Lund. Data in the figures in this paper are not presented in this order. All hospitals are teaching hospitals. The study data were retrieved from the EDs' computerized information systems during single on-site visits to each hospital in September-October 2009. In five of the six EDs quantitative data, as described below, were collected. In one of the EDs, aggregated data were obtained that enabled drilling down into accumulated data without identifying individual patients.

### Data collection and processing

The following patient visit-specific data were extracted: Patient age, time of arrival at the ED, time of first physician encounter and time of departure from the ED. There was no review of the quality of these data in this study. The throughput times length of ED stay (LOS) and time to first physician [1] were investigated as primary quality measures. In order to validate the data, the head physician, the head nurse and the data manager, or their equivalents, were interviewed concerning the data registering process. In addition, this process was scrutinized with respect to how timestamps were defined, which personnel were responsible for the data registration, and the possibility to alter data after the first registration. The definitions presented in this study comply with those recommended by Welch et al. [1] and Solberg et al. [16], and are as follows:

• Time of patient arrival at EDs A, B, D, E and F was defined as the time when the patient arrived at the reception desk. Time of patient arrival at ED C was defined as the time when the patient took a queue ticket to the reception desk.

• Time to first physician was defined as the time from patient arrival to the first registered contact with a physician providing medical assessment and/or care.

• LOS was defined as the time from patient arrival (above) to the time when the patient physically left the ED, whether discharged or admitted to in-hospital care.

The following exclusions were made in the data set in order to ensure comparability between the participating EDs and to eliminate potential data errors:

• Visits with a recorded LOS exceeding 16 hours, in most cases due to data input errors. Such visits represented 1.5% of all visits at ED C, and less than 0.3% at the other EDs.

• Visits lacking LOS data, which represented 12.6% of the visits at ED E, 2% at ED B and 0% at the other EDs.

• Visits where the patient deceased in the ED, representing less than 0.2% of the visits at all EDs.

The differences of LOS and time to first physician between hospitals were analyzed using multivariate regression analysis, with differences being considered statistically significant at p < 0.05.

### Ethics

The present study was carried out in accordance with The Declaration of Helsinki [17] and was a quality assessment initiative that included no single patients identifiable to the researchers. As such, it is exempt from review by the regional ethics committees in Sweden.

## Results

The characteristics of the participating EDs are shown in Table 1. During the study period, all EDs triaged patients into different medical specialties, so that patients were assessed by physicians from the assigned specialty. In addition, all EDs had streaming of different specific patient groups. All EDs except E had a specialist training program in Emergency Medicine (EM), but no ED had more than 1-2 EM specialists on the floor at any time. Although the IT systems in the EDs differed, there were no major differences in the data registration processes in the different EDs, and all of them provided electronic data regarding LOS, time to first physician and patient inflow patterns without significant problems.

**Table 1:** ED characteristics, in accordance with Welch et al [1]

	Emergency Department					
	**A**	**B**	**C**	**D**	**E**	**F**

**Approximate annual number of patients**	75000	65000	50000	100000	55000	70000

**Time period analysed**	Jan 1st - June 30th 2009	Jan 1st - Jun 30th 2009	Jan 1st - June 30th 2009	Jan 1st - June 30th 2009	June 8th - Oct 10th 2009	Jan 1st - June 30th 2009

**Patient visits included**	38 016	32 684	24 116	48 129	17 925	34 060

**Female patients,%**	55.4	49.6	49.3	50.4	49.6	50.7

**Admission rate,%**	23.3	26.9	46.3	34.6	Data missing	12.1

Trauma level*	1	2	1	2	1	2

**Specialties present (patient spectrum received)**	Internal Medicine, Neurology, Surgery, Urology, Orthopedics & Trauma, Infectious diseases, OB/gyn	Internal Medicine, Neurology, Surgery, Urology, Orthopedics & Trauma, Infectious diseases	Internal Medicine, Neurology, Surgery, Orthopedics & Trauma, Infectious diseases.	Internal Medicine, Neurology, Surgery, Urology, Orthopedics & Trauma, Infectious diseases	Internal Medicine, Neurology, Surgery, Orthopedics & Trauma, Infectious diseases	Internal Medicine, Neurology, Surgery, Urology, Orthopedics & Trauma, Infectious diseases

**Transplant Service in hospital**	Yes	Yes	Yes	No	Yes	Yes

**Acuity**	High	High	High	High	High	High

**EM specialist training program**	Yes	Yes	Yes	Yes	No	Yes

**IT system delivering ED data**	Take care™	Patientliggaren™, Tieto Corporation	Internally developed system	Akusys™	Cosmic™	Take care™

*trauma level according the American College of Surgeons [43]. EM, emergency medicine

**Figure 1:**
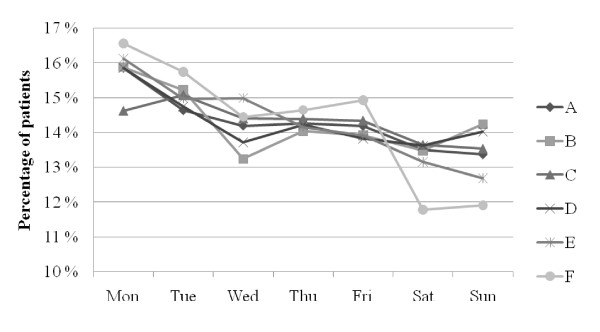
**Patient arrival to EDs A-F by day of week**.

**Figure 2:**
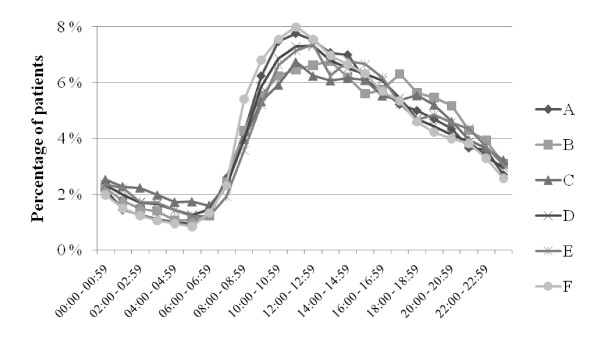
**Patient arrival to EDs A-F by time of day**.

**Figure 3:**
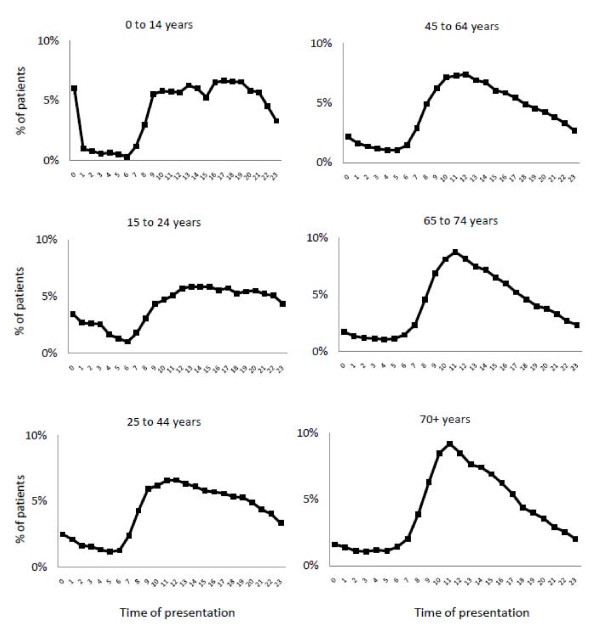
**Patient arrival to EDs by time of day and age group**.

In Figures 1, 2 and 3, ED patient inflow is presented by day of week, by time of arrival, and for different age groups. In all EDs, Monday was the busiest day (Figure 1) and Saturday was the day when the least patients arrived. The patient inflows on Wednesdays at ED B and on Saturdays at ED F were remarkably low in comparison with the other EDs. Patient inflow over the day (Figures 2 and 3) showed a homogenous pattern among the EDs. All EDs had a large increase in inflow before noon and a slow inflow decline over the rest of the 24 hour period. The noon peak and the following decline were more pronounced in older patients (Figure 3).

**Figure 4:**
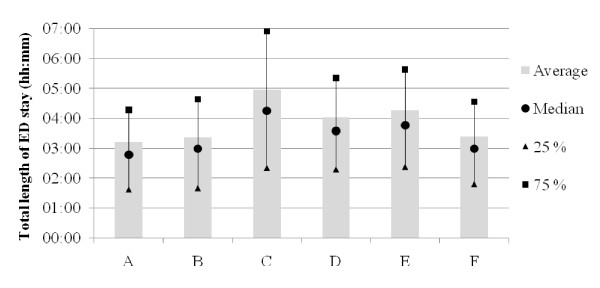
**Total length of ED stay by ED**.

**Figure 5:**
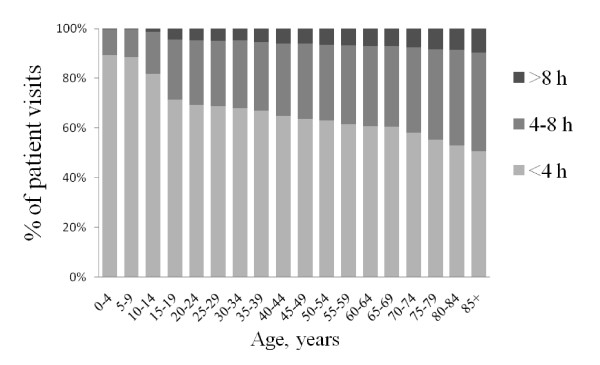
**Total length of ED stay by age group**.

**Figure 6:**
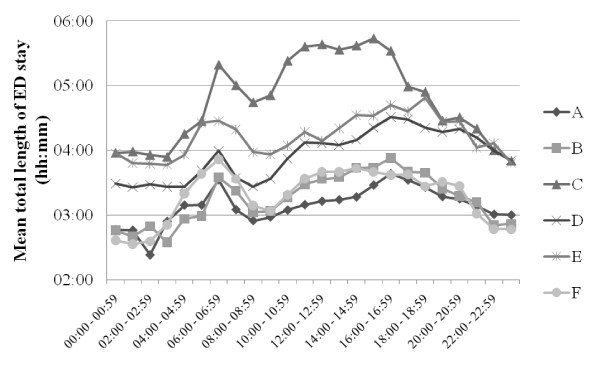
**Total length of ED stay by time of patient arrival at the different EDs**.

**Figure 7:**
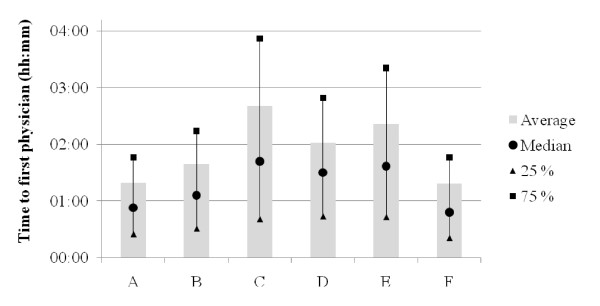
**Time to first physician by ED**.

**Figure 8:**
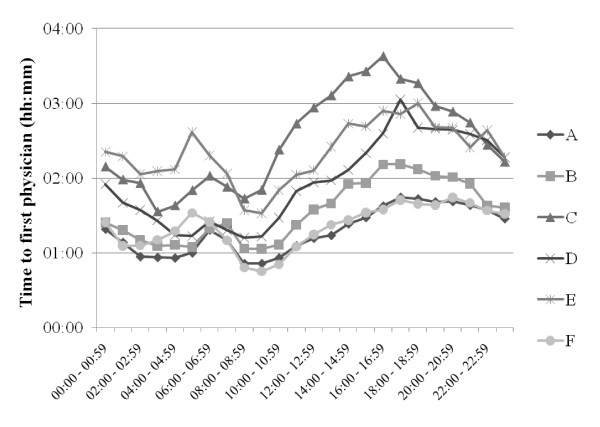
**Time to first physician by time of patient arrival at the different EDs**.

LOS data for each ED are presented in Figure 4, by age group in Figure 5, and by time of arrival in Figure 6. With the exception of ED A vs ED B (NS), all LOS differences between the EDs (Figure 4) were highly significant (p < 0.001). Average LOS was longer for older patients (Figure 5), shorter in the middle of the night (Figure 6) and clearly increased both between 6 and 7 am and in the afternoon in all EDs. The fraction of patients who were discharged from the ED within 4 hours was for ED A 71%, B 67%, C 50%, D 57%, E 54% and F 68%. Figures 7 and 8 show the time to first physician by ED (Figure 7) and by time of arrival (Figure 8). With the exception of ED A vs ED F (NS), all differences in time to physician between the EDs (Figure 7) were highly significant (p < 0.001). The time to physician (Figure 8) and the LOS (Figure 6) showed a similar diurnal pattern. In ED C, the LOS was almost 50% longer and the wait to see a physician 100% longer between 3 and 4 pm than during the early hours of the morning.

## Discussion

These results demonstrate the possibility to compile benchmarking data on quality of care markers in six different EDs in Sweden. The data presented here show that the average LOS was approximately 4 hours, of which 2 hours was spent waiting for the first physician. The throughput times in all EDs were shortest after midnight and longest in the late afternoon or early evening.

The average LOS of 4 hours and average discharge rate of 62% at 4 h in the present EDs are clearly below the quality goal set by many Swedish health care authorities of an 80% discharge rate at 4 h. According to QMAE, the average 4 h ED discharge rate in England during the same period was above 98% [18,19], which was also the national goal at the time. In the US, the median ED LOS in 2008 was 2 h and 34 min [20]. In the present study, 2 hours was instead spent waiting for the first physician, as compared to 56 min in the 2006 US NHAMCS data [21] and 77 min (first physician or nurse) in the 2009-10 UK HES data [14]. In the present study, LOS was strongly age-dependent (Figure 5), which is very similar to what has been reported from the UK [22]. Older patients stay longer in the ED.

All others things being equal, a long stay in the ED and a long wait for the physician reflects a low quality of care and decreases patient satisfaction [23]. The above comparison with UK and US throughput times supports initiatives to accelerate care in Swedish EDs. Actions to decrease process times for the elderly may be of special importance for the overall quality of care, since they are a significant proportion of the patients (e.g. [24]) and on average are more likely to suffer from long waiting times. Interestingly, the relative differences in LOS between the EDs (Figure 4) were similar to the differences in physician waiting times (Figure 7), indicating that that they are linked. Indeed, it seems likely that a short wait for the critical decision-maker, the physician, will increase the chances of a short LOS. The reasons for the long throughput times in the present EDs are however most likely multiple, and probably include slow turnaround times for blood samples, radiology exams and admissions, a relative lack of personnel and, most importantly, an ineffective organization.

In Sweden, like in Norway, Denmark and Finland, ED patients are usually sorted into medical specialties by a triage nurse, and then managed by physicians from the respective specialties, most often internal medicine, surgery and orthopedic surgery. We believe that introducing more EM specialists would simplify and increase the flexibility of the ED organization and that this in turn would probably enhance patient throughput. Other solutions that have been proposed for long throughput times include streaming of patients with less severe illnesses into fast tracks [25,26], point-of-care testing [27,28], nurse practitioners in the ED [29], nurse-requested X-ray [30,31] and team triage [32,33]. For most of these methods however, adequate studies regarding their precise effects are lacking [34].

The throughput times in this study varied with the time of patient presentation in all EDs, with the largest variation in ED C. LOS in EDs C and F was markedly increased at lunchtime and almost stable during the afternoon, whereas in all other EDs, LOS increased over the afternoon (Figure 6). The reasons for the patterns in EDs C and F are unclear, but according to the leadership in ED C, the pattern in ED C may be related to hospital crowding with admitted patients waiting in the ED for an in-hospital bed. The LOS pattern in ED C and F is an example of a finding that will be useful for the individual ED to analyze further, e.g. by using the conceptual models suggested by Asplin et al. [35,36]. A long LOS was observed in all EDs when the patients arrived between 6 and 7 am. This was most likely caused by patient handovers between the night and day shifts and could thus be influenced by organizational changes.

The observed diurnal variation in LOS and waiting times in all EDs is most likely due to a mismatch between allocated resources and patient inflow over the 24 hours, with a relative excess of personnel and resources during the night. One explanation of this excess may be the lack of an EM physician-based organization with a consequent need for more doctors (from multiple specialties) to cover the spectrum of ED patients at night. This is supported by data from UK, where LOS in EDs with EM physicians is instead longer during the night than during the day [14,22], and where this has been explained by a lower physician staffing at night than would be possible in Swedish EDs [22].

The circadian pattern of ED patient inflow in this study (Figure 2) was similar to that shown repeatedly in the UK [14,15,37] and the US [21]. Also, the impact of age on the pattern of presentation (Figure 3) was remarkably similar to that in UK reports [37]. This stability over time and between age groups and EDs with different organizational structures indicates that patient inflow is little affected by the emergency health care system, and that initiatives to change inflow are unlikely to be successful. Instead, the ED organization needs to be adapted to meet the inflow at hand. Published models to forecast patient inflow [38] may be used as aids. The different inflow patterns in the different age groups (Figure 3) may be of importance for the distribution of specific ED resources during the day.

As in UK EDs [37], and in contrast to US EDs [38], Saturdays was a low inflow day in the present EDs. The reason for this difference is unclear and warrants further research.

### Limitations

The participating EDs are all adult EDs in university hospitals and therefore the results are not necessarily generalizable to smaller units, or to EDs receiving children primarily.

In all but one ED (C), the throughput times were calculated from the first registration by the personnel, and not from the actual time of patient arrival. Since there is often an interval between arrival and registration, the "real" LOS for all EDs except in C were somewhat longer than described in the results. Data from the Skåne University Hospital ED in Malmö suggest that this interval is on average some 15 min [39]. EDs A, B and D-F have recently changed to measuring LOS from the actual time of patient arrival, ie the taking of a queue ticket.

The medical specialties were not similar in the EDs (Table 1), and since some specialties have shorter LOS and waiting times than others, these differences may have influenced the results.

### Development of ANSWER

When fully developed, ANSWER will encompass the entire pre- and in-hospital emergency care system in Sweden (approximately 2 million patients/year [40]) so that near-real time data from all participating institutions are available for quality improvement, epidemiology, disease control and public health surveillance. The large number of observations will decrease the influence of chance on the results, and the ANSWER data will thus also be useful for research projects. ANSWER data may perhaps even be used as a surrogate for randomized controlled trials, which are often difficult to conduct in EM. There are 71 national health care quality registries receiving public financial support in Sweden [41], and this abundance provides excellent opportunities for data linking and collaboration.

ANSWER will collect data for all ED patients as a first step in its development. Automated data capture from the patient records through XML files will be used and allows near-real time surveillance, close to complete patient coverage and minimal selection bias. In addition to patient characteristics, the data variables to be collected are chosen to reflect the quality of ED care as defined by the Swedish Board of Health and Welfare [42]. The variables include chief complaints, throughput times (LOS, time to physician, discharge to physically leaving the ED etc), ED and hospital stay diagnoses, mortality in the ED, and morbidity and mortality within 30 days. Information on the patient's experience of the visit is also of interest, but a system for the collection and automatic inclusion of such data remains to be developed. In addition, ANSWER like the NC DETECT [9,10] will face the challenge of establishing a standard list of specific terms for the chief complaint, and also of triage priority levels. The UK HES and QMAE data do not include a variable for chief complaint.

## Conclusions

This study demonstrates the feasibility of collecting benchmarking data in emergency care in Sweden, and forms the basis for ANSWER. In the studied six EDs, Monday was the busiest and Saturday the least busy day. All EDs had a large increase in patient inflow before noon and a slow decline over the rest of the 24 hours. The average length of stay was 4 hours of which 2 hours was spent waiting for the first physician. These quality measures showed a considerable diurnal variation. ANSWER aims to become a Swedish national quality registry for all emergency care, and one of its strengths will be the automated data capture from participating centers. By providing reliable benchmarking data, we believe that ANSWER will facilitate systematic quality improvement in the emergency care process, organizational planning, and research in EM.

## Competing interests

The authors declare that they have no competing interests. FE and PT are employed by Nordic Healthcare Group, NHG. NHG is a commercial company that focuses on healthcare and welfare industries and designs models to enhance productivity, cost-effectiveness and process quality. The business is based on research and has employees in Stockholm, Sweden and Helsinki, Finland.

## Authors' contributions

UE participated in the conception and design of the study, data interpretation and drafted and critically revised the manuscript. LK, AL, PL and MC participated in the conception and design of the study, data interpretation and critically revised the manuscript. FE and PT collected and analyzed the data and critically revised the manuscript. FE also drafted the manuscript. All authors read and approved the final version of the manuscript.
